# Supplementary material for: Thermalization and criticality on an analogue–digital quantum simulator
Source: Nature. 2025 Feb 5;638(8049):79–85. doi: 10.1038/s41586-024-08460-3 (PMC11798852; doi:10.1038/s41586-024-08460-3)
Supplement: Supplementary file 1 — The Supplementary Information includes Notes 1–13 and Figs. 1–16. In this file, we describe MPS simulations of XY model dynamics, numerical finite-size scaling analysis, alternative correlation fitting schemes and further theoretical analysis of XEB experiments, including computational complexity. [file 41586_2024_8460_MOESM1_ESM.pdf]

nature portfolio
https://doi.org/10.1038/s41586-024-08460-3

Supplementary information

Thermalization and criticality on an analogue–digital quantum simulator

In the format provided by the authors and unedited

SUPPLEMENTARY MATERIALS

S1. MPS simulations of Kibble-Zurek and diffusion experiments

To classically compute the time evolution of the state for the Kibble-Zurek experiments with $N_\mathrm{q} = 65$ sites, we perform time-dependent matrix product state simulations. Concretely, we employ the time-dependent variational principle (TDVP)[82] with two-site update to capture the buildup of bond dimension necessary when starting from the initial product state. We use the full Hamiltonian describing the experimental setup including next-nearest neighbor couplings and three-qubit terms illustrated in Extended Data Fig. 4. As a time step in the simulations, we choose $t_\mathrm{r}/1000$, which translates to a maximal time step of 0.0625 in units of $1/g_\mathrm{m}$ for the longest ramp times.

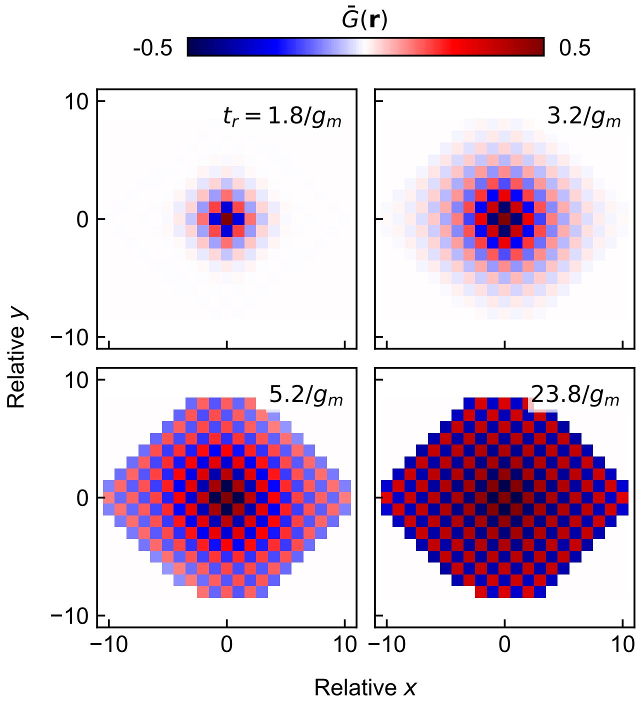

Fig. S1. **Averaged correlations at different ramp times.** Simulated equivalent of Fig. 3c of the main text showing the increase of the average correlations $\bar{G}(\mathbf{r})$ with increasing ramp time.

This, on the one hand, ensures the absence of any significant error due to the finite time step, and, on the other hand, gives identical discrete increments of the time-dependent Hamiltonian along the ramp for all t_r. We simulate the dynamics for bond dimensions of up to $\chi = 1024$ which leads to sufficient convergence of the observables measured in the experiment. The ability to obtain accurate results using MPS simulations for this large system size benefits from the interplay of two factors. If the ramp time is short, the system has only limited time to generate entanglement. If the ramp time is longer, the time-evolved state remains close to the ground state, guaranteeing area-

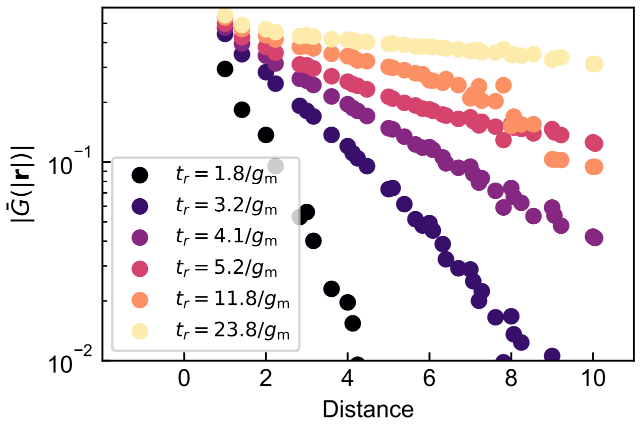

Fig. S2. **Averaged correlations as a function of distance at different ramp times.** The correlations at different distances agree well with the experimental data shown in Fig. 3d. As in the experiment, there is a crossover between exponential decay at small ramp times and power-law behavior at large t_r.

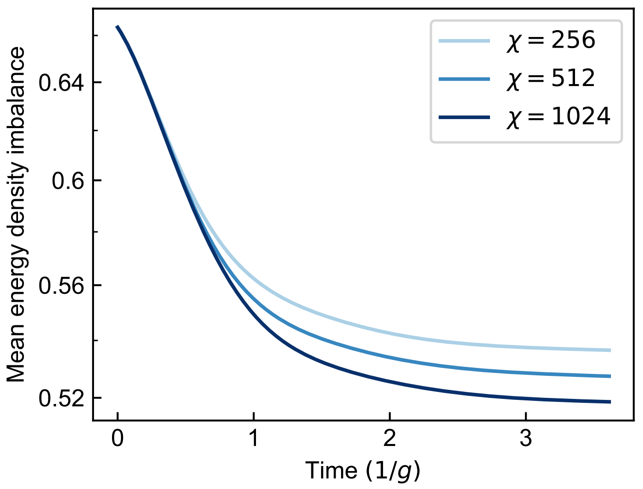

Fig. S3. **Breakdown of MPS simulations for energy diffusion.** The energy imbalance between the halves of the system prepared in high and low energy dimer coverings, respectively, first falls off exponentially as expected by diffusion (axis in log scale), but then plateaus due to the lack of bond dimension to faithfully represent the time-evolved state. The simulation already fails to capture the energy evolution at a time of $\sim 1/g$ at the highest bond dimension of $\chi = 1024$.

law behavior with a logarithmic correction[55]. The growth of entanglement is limited in both cases, making the MPS approach ideal to simulate this set of experiments.

As already presented in the main text for a number of observables, this leads to a good agreement between experiment and simulation. Here, we present some additional numerical data. In Fig. S1, we show the simulated version of Fig. 3c of the main text which agrees well with the experimental data. The behavior of $\bar{G}(\mathbf{r})$ as a function of distance is depicted in Fig. S2. We observe very accurate agreement with experiment for smaller ramp times. For larger ramp times, the longer-range correlations in the

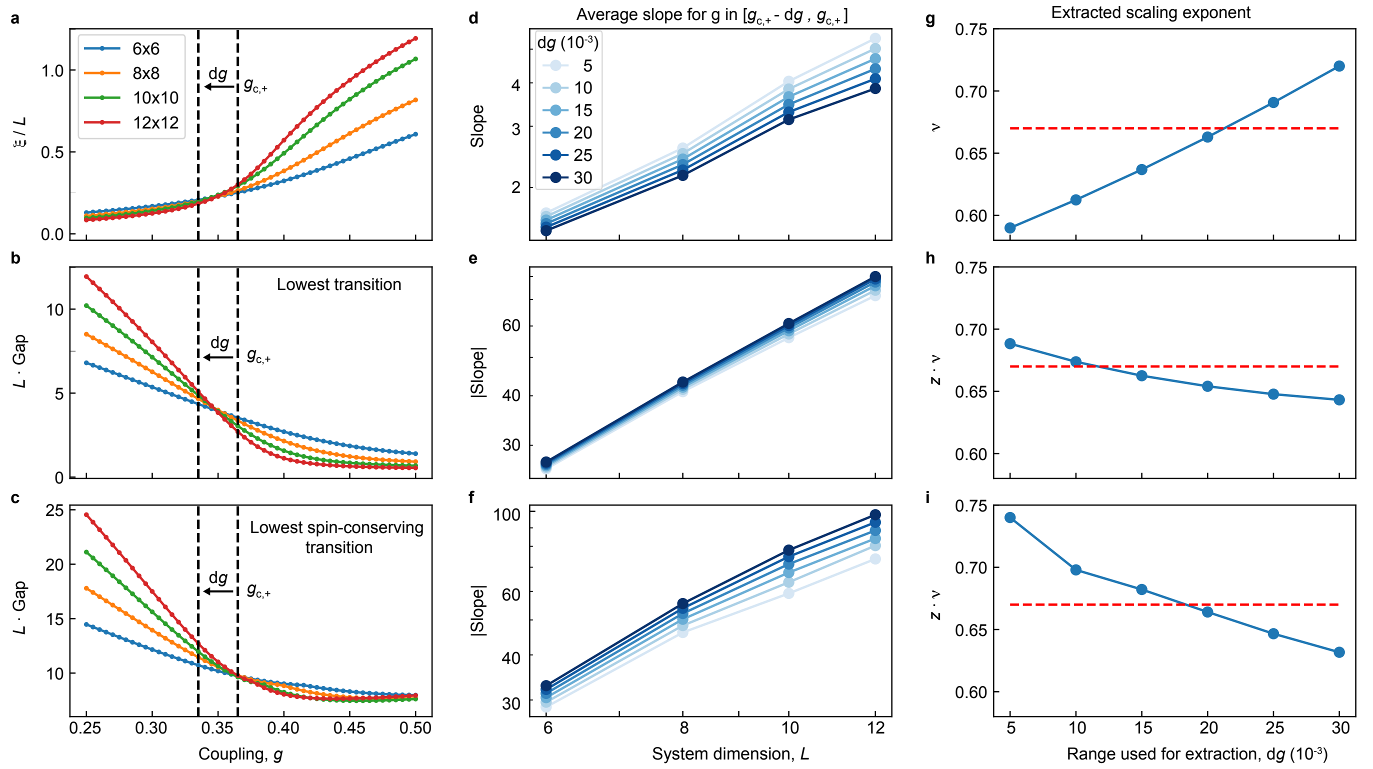

Fig. S4. **Numerical extraction of scaling exponents through finite-size scaling analysis.** **a,** Correlation length of ground state normalized by system dimension L, determined from MPS simulations and plotted versus coupling, g (see text for further details). Finite-size crossing is observed near $g_c = 0.35$. Black dashed vertical lines indicate extrema of ranges used for slope extraction. **b,c** Same as **a**, but for gap multiplied by system dimension. We consider both the overall lowest transition (**b**) and the smallest spin-conserving one (**c**). **d-f,** Extracted slopes from **a-c**, respectively, for ranges of various widths, and plotted as a function of system dimension, L. The slopes grow near-algebraically with L, as expected. **g-i,** Extracted scaling exponents from **d-f**, respectively. In all cases, the extracted exponents are consistent with the predicted $z = 1$, $\nu = 0.67$ (red dashed line).

experiment are slightly suppressed due to decoherence, while they become almost constant in the simulation.

We also attempted to simulate the energy transport experiment in which two halves of the system are prepared with high and low energy dimer covering. The average energy imbalance between the left and right parts of the system is depicted in Fig. S3. Due to the large buildup of entanglement in this case, which should be contrasted with the Kibble-Zurek experiments, the MPS method is severely limited and cannot capture the energy diffusion across the system.

S2. Numerical finite-size scaling analysis

We here perform finite-size scaling analysis to evaluate the critical scaling exponents ν and z near the critical point in the XY-model studied in our work. We perform DMRG simulations on square-shaped clusters of $L \times L$ sites to determine the correlation length of the ground state (Fig. S4a), as well as the gap size, Δ, of both the lowest transition ($S_z = 0 \to S_z = 1$; Fig. S4b) and the lowest spin-conserving transition ($S_z = 0 \to S_z = 0$; Fig. S4c), while sweeping the staggered field (h) from 1 to 0 and the coupling (g) from 0 to 1. We use a bond dimension $\chi = 2048$ and system sizes $L \times L$ with $L \in \{6, 8, 10, 12\}$. Near the critical point, it is expected that $\xi/L = F_\chi((g - g_c)L^{1/\nu})$ and $L\Delta = F_\Delta((g - g_c)L^{1/(z\nu)})$. Motivated by this, we plot ξ/L and $L\Delta$, and observe finite-size crossing near $g_c = 0.35$. As can be seen from the expressions above, the slope near the critical point is expected to scale as $L^{1/\nu}$ and $L^{1/(z\nu)}$ for the correlation length and gaps, respectively. Hence, to evaluate the scaling exponents while also accounting for variations in slope near the critical point, we extract the slope in ranges of varying width dg from $g_{c,+} - dg$ to $g_{c,+}$ with $g_{c,+} = 0.365$ (Figs. S4d-f). As theoretically expected, we find that the slopes increase near-algebraically with L, from which we extract scaling exponents shown in Figs. S4g-i. For all three cases, we find that the extracted exponents are consistent with the expected $\nu = 0.67$, $z = 1$.

S3. Further comparison of coarsening observations with literature

In our study of the scaling of the correlation length with ramp time, we observe clear discrepancies from the predictions of the Kibble-Zurek mechanism, attributed to coarsening dynamics in our system (Fig. S5a). Here we

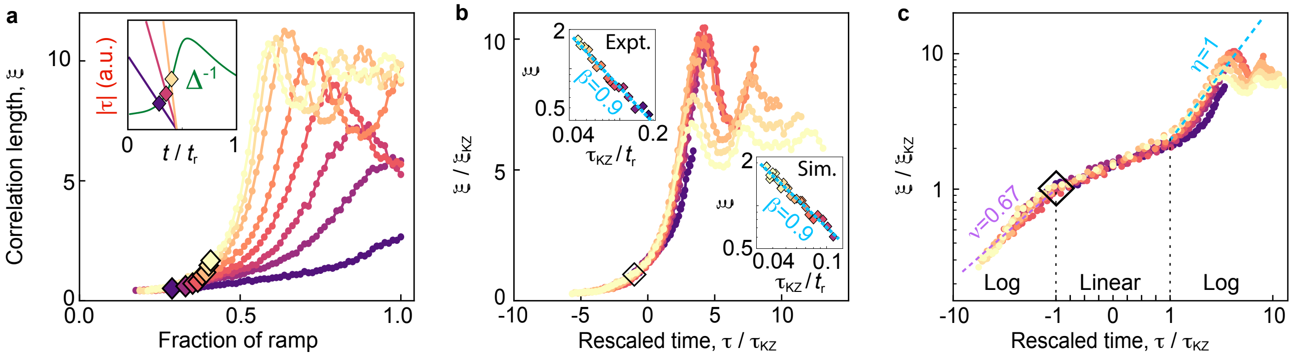

Fig. S5. **Dynamical universality driven by coarsening: a,** Correlation length as a function of time along the ramp for various ramp durations. Diamonds represent expected freezing point, at which $\Delta^{-1} = |\tau| = |t - t_c|$ (inset). **b,** Correlation length rescaled by ξ_{KZ}, plotted against the rescaled time for various ramp durations (colored dots), leading to a notable collapse. Insets: Measured (upper left) and simulated (lower right) correlation length at the theoretically predicted freezing point, displaying power-law behavior, $\xi(\tau = \tau_{\mathrm{KZ}}) = \xi_0(\tau_{\mathrm{KZ}}/t_r)^{-\beta}$ with $\beta = 0.9(1)$. **c,** Same as **a**, but with two-sided logarithmic axes for $\tau < -\tau_{\mathrm{KZ}}$ and $\tau > \tau_{\mathrm{KZ}}$. In the first regime, the data shows similar behavior to the theoretically expected scaling, $f(x) = |x|^{-\nu}$ with $\nu = 0.67$ (purple dashed line). In the second regime, we find an increase $f(1)/f(-1)$=2.3. In the third regime, we observe power-law-like behavior with a (heuristic) exponent near $\eta = 1$.

discuss how these observations compare with theoretical predictions of universal coarsening behavior near a critical point, and refer to Refs.[5] and [21] (on which this discussion is based) for even further detail. As in the main text, we define the freezing time, t_{KZ} as the time at which the inverse gap starts exceeding the remaining time to the critical point, $\Delta(t = t_{\mathrm{KZ}})^{-1} = t_c - t_{\mathrm{KZ}} \equiv \tau_{\mathrm{KZ}}$ (inset of S5a). We find that the correlation length at this time scales as a power-law, $\xi_0(\tau_{\mathrm{KZ}}/t_r)^{-\beta}$, with a somewhat larger exponent $\beta = 0.9(1)$ than the theoretical prediction, $\beta = \nu = 0.67$, for infinite system size (inset of Fig. S5b). This is also observed in MPS simulations and is expected to be due to finite-size effects.

It was theoretically predicted that rescaling of time and the correlation length by τ_{KZ} and ξ_{KZ}, respectively, should reveal universal coarsening behavior in which the dynamics become insensitive to the ramp time, i.e. $\xi/\xi_{\mathrm{KZ}} = f(\tau/\tau_{\mathrm{KZ}})$. Indeed, we observe such a collapse of the curves collected for different ramp times in Figure 3 in the main text (also in Fig. S5b), consistent with this prediction. We emphasize that the rescaling involves only the single parameter, ξ_0, extracted from fits of $\xi(\tau = \tau_{\mathrm{KZ}} = \xi_0(\tau_{\mathrm{KZ}}/t_r)^{-\beta}$, rather than manually enforcing that $\xi(\tau = \tau_{\mathrm{KZ}})/\xi_{\mathrm{KZ}} = 1$ by using the experimentally observed $\xi(\tau = \tau_{\mathrm{KZ}})$, thus making the collapse even more convincing.

To further understand the functional form of f, it is convenient to divide the Hamiltonian ramp into three sections (Fig. S5c), namely 1) the adiabatic regime ($\tau \ll -\tau_{\mathrm{KZ}}$), 2) the critical regime ($-\tau_{\mathrm{KZ}} < \tau < \tau_{\mathrm{KZ}}$), and 3) the non-critical regime ($\tau \gg \tau_{\mathrm{KZ}}$). In the adiabatic regime, the system is expected to remain near the ground state and thus exhibit a correlation length given by $\xi(\tau) = \xi_{\mathrm{KZ}}|\tau/\tau_{\mathrm{KZ}}|^{-\nu}$, or $f(x) = |x|^{-\nu}$. We observe behavior similar to this prediction, with only small deviations that we attribute to the finite size of our system. In the second (critical) regime, it is less trivial to predict f with a simple functional form, but the growth of ξ is expected to be of order unity. In the experiment, we find that the collapsed curves exhibit $f(1)/f(-1) = 2.3 \pm 0.2$, but note that this is the regime in which finite size effects are expected to be of most significance. Finally, in the non-critical regime in the ordered phase, the curves collapse very well all the way up to $\tau/\tau_{\mathrm{KZ}} = 3$, showing power-law-like behavior with an exponent close to 1. Importantly, in this regime, there is a crucial difference between our system and quantum Ising models which have typically been considered in this context. While a gap opens on the ordered side in the latter case, the XY-model hosts coexisting gapped and gapless modes due to spontaneous breaking of U(1) symmetry. This is likely to cause differences in the coarsening dynamics, which still remain to be fully understood.

S4. Combined exponential and power-law fitting.

We here perform fitting of the data in main text figure 3 with combined exponential and power-law fits on the form $\alpha e^{-d/\xi} d^{-\gamma}$ (Fig. S6a), which support the insights from the individual fits. As seen in Fig. S6b, the extracted correlation length from the exponential factor exhibits an abrupt increase in the same region as where the other KT-signatures are observed, indicating that the power-law behavior takes over. Moreover, in the same regime, the power-law exponent is found to reach a value even closer to 0.25 than in the individual fits (Fig. S6c). Finally, we compare the relative dominance of the two behaviors in Fig. S6d, by plotting their decay contributions over the fit range ($d_{\min} = 1$, $d_{\max} = 6$):

$$\text{Exp. contribution: } A^{-1}\left(1 - \exp\left[(d_{\min} - d_{\max})/\xi\right]\right), \quad (1)$$

$$\text{Power-law contribution: } A^{-1}\left(1 - (d_{\min}/d_{\max})^{\gamma}\right), \quad (2)$$

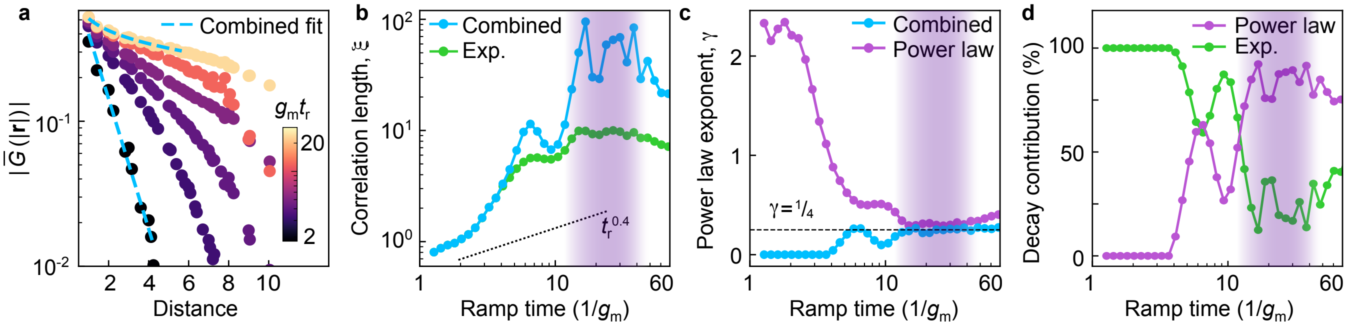

Fig. S6. **Combined exponential and power-law fits. a,** Distance-dependence of correlations for various ramp times, fit with combined exponential and power-law fits (teal dashed lines). **b,c** Ramp time dependence of correlation length (**b**) and power-law exponent (**c**), extracted from combined (teal), exponential (green) and power-law (purple) fits. **d,** Decay contributions, as defined in the text, for power-law (purple) and exponential (green) fits.

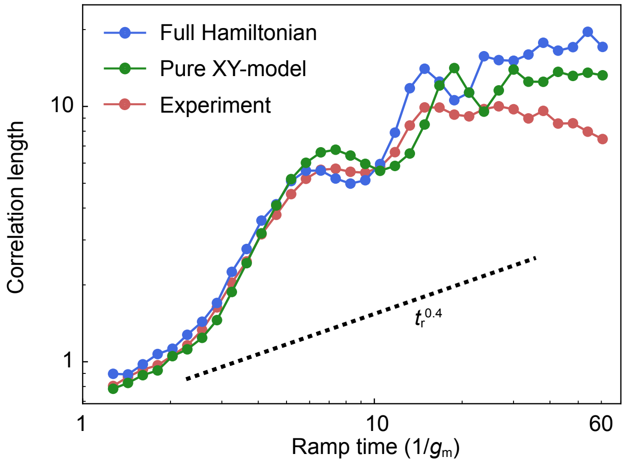

Fig. S7. **Comparison of device Hamiltonian and pure XY-model.** MPS simulation of the ramp time dependence of the correlation length for the device Hamiltonian (blue) and the pure XY-model (green), showing very similar behavior and a clear deviation from Kibble-Zurek scaling (dashed black) in both cases.

where $A = 1 - \exp\left[(d_{\min} - d_{\max})/\xi\right](d_{\min}/d_{\max})^{\gamma}$ is the total decay. Consistent with the conclusions drawn from the individual fits, we find that the decay contribution from the power-law strongly overtakes that of the exponential in the same low-temperature regime as where the other signatures are observed.

S5. Comparison of pure XY-model with device Hamiltonian

Our new calibration scheme allows us to accurately estimate the higher order terms in our Hamiltonian, the largest of which are typically about 5-10 times smaller than the hopping term. In Fig. S7, we compare MPS simulations of the correlation length for the actual device Hamiltonian and the pure XY-model. Importantly, we find very similar qualitative behavior and a clear breakdown of the Kibble-Zurek mechanism for both models.

S6. Empirical estimation of self-XEB

1. *Ideal case*

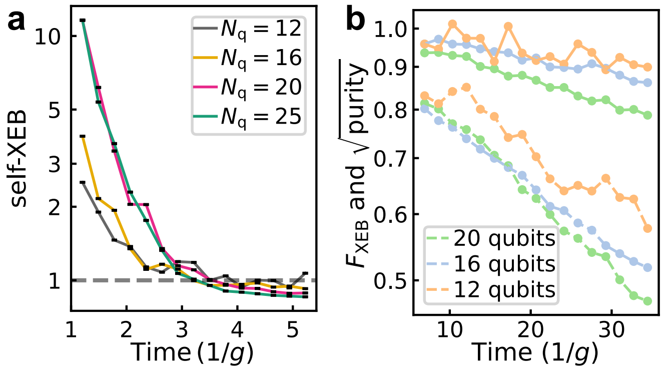

Fig. S8. **Purity from self-XEB. a,** Experimental self-XEB estimated empirically from experimental data using the unbiased estimator Eq. (5). The black markers show error bars. **b,** Solid lines show the estimate of the $\sqrt{\text{purity}} = \Phi$ assuming the global depolarizing channel Eq. (6) using the expression Eq. (8). The dashed lines show the XEB Fidelity F_{XEB} obtained from the experimental data used in the main text.

In the main text, we measure self-XEB by constructing an empirical bitstring distribution $p_{\text{exp.}}(s_i) = \frac{M_i}{M}$, where M_i is the total number of times a bitstring s_i was sampled from a quantum device (after postselecting bitstrings with the correct number of excitations), and M is the total number of postselected bitstrings. An empirical estimate for the self-XEB reads

$$\text{self-XEB}_{\text{est.}} = D \sum_{s_i} \frac{M_i^2}{M^2} - 1, \tag{3}$$

and M_i follows the binomial distribution $P(M_i = x) = \binom{M}{x} p_i^x (1-p_i)^{M-x}$, where p_i is the quantum probability of sampling a bitstring s_i. The second moment of the binomial distribution reads $\mathbb{E}\, M_i^2 = M^2 p_i^2 + M p_i (1-p_i)$,

therefore

$$\mathbb{E}\,\text{self-XEB}_{\text{est.}} = \text{self-XEB}_{\text{true}} + \frac{D}{M}\sum_{s_i} p_i(1-p_i) = \quad (4)$$

$$= \text{self-XEB}_{\text{true}} + \frac{D}{M} - \frac{1}{M}\left(\text{self-XEB}_{\text{true}}+1\right) =$$

$$= \left(1-\frac{1}{M}\right)\times \text{self-XEB}_{\text{true}} + \frac{D-1}{M},$$

which means that self-XEB$_{\text{est.}}$ is a biased estimator with the relative bias of $1/M$ and the absolute bias of $(D-1)/M$. To account for this, in the main text, we estimate self-XEB using an unbiased estimator

$$\text{self-XEB}_{\text{est., unbiased}} = \frac{\text{self-XEB}_{\text{est.}}}{1-1/M} - \frac{D-1}{M-1}. \quad (5)$$

2. Depolarizing channel

Study of the bitstring distribution provides information about incoherent errors under some assumptions on the noise type[44]. For simplicity, we consider a global depolarizing channel, which modifies bitstring probabilities as

$$\tilde{p}_i = \Phi p_i + (1-\Phi)/D, \quad (6)$$

where p_i is a bitstring probability in the ideal case, and $\Phi^2 =$ purity. This approximation yields

$$\text{self-XEB}_{\text{noisy}} = D\sum_i \tilde{p}_i^2 - 1 = \Phi^2 \times \text{self-XEB}_{\text{ideal}}. \quad (7)$$

Therefore, purity may be estimated as

$$\Phi^2 = \text{self-XEB}_{\text{noisy}}/\text{self-XEB}_{\text{ideal}}, \quad (8)$$

where self-XEB$_{\text{ideal}} = D\sum_i p_i^2 - 1$ is estimated using an ideal classical simulation and quickly converges to a ~ 1 value as shown in Appendix S13, and self-XEB$_{\text{noisy}}$ is estimated empirically from experimental data using the unbiased estimator Eq. (5) and is shown in Fig. S8a. With this benchmark, we get an independent estimate of the system's incoherent errors. The result is shown in Fig. S8b. We conclude that the error rates quoted in the main text are mainly dominated by coherent sources. We note that although the decoherence processes in this experiment are mainly due to dephasing (since postselection eliminates decay processes), we expect the simplified depolarizing-channel model to correctly capture the magnitude of incoherent processes.

S7. Exact state-vector simulation

The time evolution performed in the experiment consists of a short time-dependent on-ramp, a long time-independent "plateau", and a short time-dependent off-ramp. To simulate the time-dependent ramps, we solve the time-dependent Schrödinger equation using the `Runge-Kutta-45` algorithm.

For stationary evolution, we employ the Chebyshev polynomials approach[59,60]. For $x \in [-1,1]$ and any real τ, an exponential can be decomposed as

$$e^{-ix\tau} = \sum_{m=0}^{+\infty} \alpha_m (-i)^m J_m(\tau) T_m(x), \quad (9)$$

where $\alpha_0 = 1$, $\alpha_m = 2$ for $m > 0$, $J_m(\tau)$ is a Bessel function of the first kind of the m-th order, and $T_m(x) = \cos[m\arccos(x)]$ is the m-th Chebyshev polynomial. These polynomials obey the following recurrence relation:

$$T_{m+1}(x) + T_{m-1}(x) = 2\,x\,T_m(x), \quad (10)$$
$$T_0(x) = 1,\ T_1(x) = x.$$

To time-evolve a wave function $|\psi(0)\rangle$ for a time t with a Hamiltonian $\hat{H}$, we need to apply the Chebyshev decomposition Eq. (9) to the matrix exponential $\exp\left(-it\hat{H}\right)$. To this end, we introduce a rescaled Hamiltonian

$$\hat{h} = \frac{1}{E_{\max}-E_{\min}}\hat{H} - \frac{E_{\max}+E_{\min}}{2(E_{\max}-E_{\min})}\hat{I}, \quad (11)$$

where $\hat{I}$ is the identity operator, and $E_{\min}, E_{\max}$ are the minimum and maximum eigenvalues. The resulting rescaled Hamiltonian $\hat{h}$ has its spectrum within $[-1,+1]$, as the decomposition Eq. 9 requires. In practice, it is sufficient to only set an upper bound on the bandwidth $W = (E_{\max}-E_{\min})$ to ensure and all eigenspectrum lies within $[-1,+1]$. Applying a corresponding rescaling of the evolution time $\tau = t\times(E_{\max}-E_{\min})$, we obtain

$$|\psi(t)\rangle = J_0(\tau)|\psi_0^{\hat{h}}\rangle + 2\sum_{m=1}^{+\infty} J_m(\tau)|\psi_m^{\hat{h}}\rangle, \quad (12)$$

where we defined the Chebyshev partons:

$$|\psi_{m+1}^{\hat{h}}\rangle = (-i)^{m+1} T_{m+1}(\hat{h})|\psi(t=0)\rangle = \quad (13)$$
$$-2i\hat{h}|\psi_m^{\hat{h}}\rangle + |\psi_{m-1}^{\hat{h}}\rangle,$$
$$|\psi_0^{\hat{h}}\rangle = T_0(\hat{h})|\psi(t=0)\rangle = |\psi(t=0)\rangle,$$
$$|\psi_1^{\hat{h}}\rangle = (-i)T_1(\hat{h})|\psi(t=0)\rangle = -i\hat{h}|\psi(t=0)\rangle.$$

For $\|\hat{h}\| < 1$, all Chebyshev polynomials are bounded, $\|T_m(\hat{h})\| < 1$, which guarantees stability and convergence of the algorithm. To obtain the required number of matrix-vector operations, we consider large-m asymptotics of the Bessel functions for $\tau \ll \sqrt{m+1}$:

$$J_m(\tau) \sim \frac{1}{\Gamma(m+1)}\left(\frac{\tau}{2}\right)^m \sim \frac{1}{\sqrt{2\pi m}}\left(\frac{e\tau}{2m}\right)^m, \quad (14)$$

which makes this Bessel function reach a maximum at $m = m^*/e = \tau/2$ and then decay super-exponentially with m for $m > m^* = e\tau/2 = (e/2)\times(E_{\max}-E_{\min})t$.

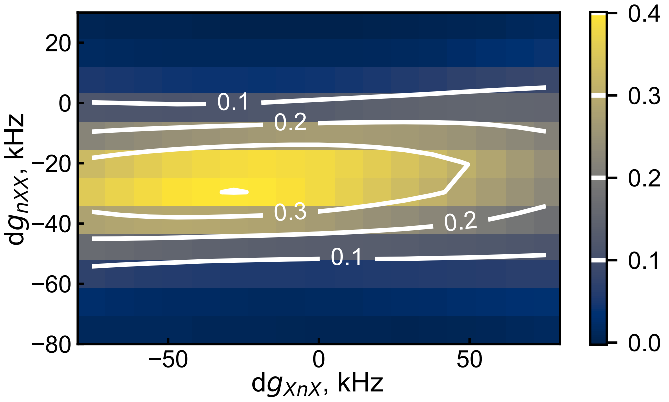

Fig. S9. **Optimizing the XEB fidelity.** Here, we show XEB fidelity for different values of the uniform global shifts in g^{nXX} and g^{XnX}. We consider the data from the main text with the system size of 25 qubits. The scan suggests an optimum shift at $\mathrm{d}g^{nXX} \approx \mathrm{d}g^{XnX} \approx -20\,\mathrm{kHz}$.

Thus, the threshold m^* defines the typical number of matrix-vector actions that are necessary to time-evolve a wave function for time t. Therefore, we see that the Chebyshev time evolution algorithm complexity is linear in the evolution time, the Hamiltonian bandwidth, and Hilbert space dimension, $\mathcal{O}(DtW)$.

The recurrence relation Eq. 13 requires storing 4 vectors. We stop iterating when the norm of the time-evolved state satisfies $|\||\psi(t)\rangle\|-1|< 10^{-10}$. Finally, given the time stamps $t_0, t_1, \ldots$ used to produce experimental bitstring samples, we time-evolve our wave function consecutively between these time stamps, setting $t = t_{i+1} - t_i$ and starting from a previously obtained wave function.

To perform these simulations, we use the `lattice-symmetries` package[61], which utilizes excitation number conservation and matrix-less matrix-vector operations. A single `c3d-standard-360` node with 1.4 Tb RAM on the Google Cloud Platform allows to exactly (up to the 10^{-10} stopping criterion error) time-evolve a wave function with the Hilbert space dimension of $\sim 9 \times 10^9$ for $t \times (E_{\max} - E_{\min}) = 2 \times 10^3$ in only 5 days. This Hilbert space size corresponds to 18 excitations in a 36-qubit system, or to 8 excitations in a 64-qubit system.

S8. Few-parameter XEB optimization

In the calibration protocol used in this work, we produce terms in the effective spin-Hamiltonian $\hat{H}_{\mathrm{s}}$ by considering sub-systems of the chip. Therefore, a small possible bias in this procedure (due to the patch Hilbert space truncation) would be replicated in the whole system and cause coherent errors[62]. We ameliorate this effect by performing *a posteriori* XEB optimization over only two global parameters, namely uniform shifts in the couplings g^{XnX} and g^{XXn} defined in Eq. (3) (Fig. S9). All other global shifts do not improve the XEB. This two-parameter fit finds very small optimal shifts of $\mathrm{d}g^{XnX} \approx \mathrm{d}g^{nXX} \approx -20\,\mathrm{kHz}$, which are optimal for all system sizes and occupations, thus confirming our assumption about the systematic nature of these coherent errors.

S9. Fidelity prediction at a given system size and time

In this work, time evolution consists of a fast on-ramp, time evolution with a Hamiltonian H_s over a much longer time, and a fast off-ramp. We write an ansatz for XEB fidelity expected after a fixed evolution time t with H_s on a system with size N_{q}:

$$F(t, N_{\mathrm{q}}) = F_0^{N_{\mathrm{q}}} e^{-\epsilon \times N_{\mathrm{q}} \times (t/T)}, \tag{15}$$

where ϵ is the per-qubit-per-cycle error, T is the cycle time, and F_0 accounts for fidelity loss during a 6-ns on-ramp, 6-ns off-ramp, and readout. We fit the XEB fidelities obtained in the dataset from the main text with system sizes from 12 to 35 qubits, and obtain $F_0 = 0.9946$, $\epsilon = 9.4 \times 10^{-4}$. The resulting fits are shown in Fig. S10, and the fit yields root-mean-square-error of 2.4×10^{-3}.

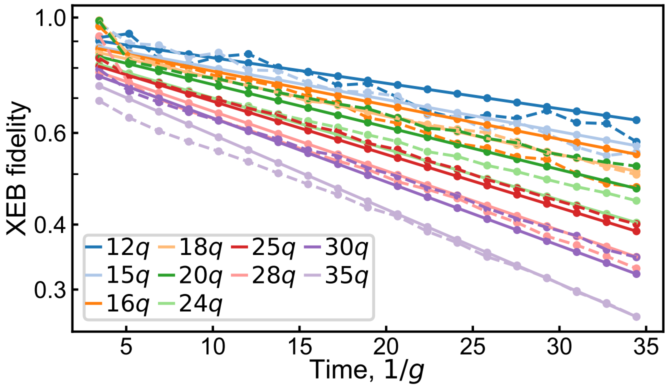

Fig. S10. **Fitting the XEB fidelity** Solid lines are the experimental results also shown in the main text, and the dashed lines show a global fit using Eq. (15).

S10. Entanglement of time-evolved states

In this section, we study the growth of entanglement in our system, and the evolution of its Schmidt spectrum. Most of the computations are performed using exact state-vectors, and some simulations employ matrix-product states. Most importantly, we analyze the effects of particle-number conservation on the entanglement properties.

1. *Schmidt values distribution*

For all the (rectangular) geometries, we compute entanglement entropy between two parts of the N-qubit system of sizes $L = \lfloor N/2 \rfloor$ and $R = N - L$. The left part of the system includes all sites with $x + y \times L_x < L$, where

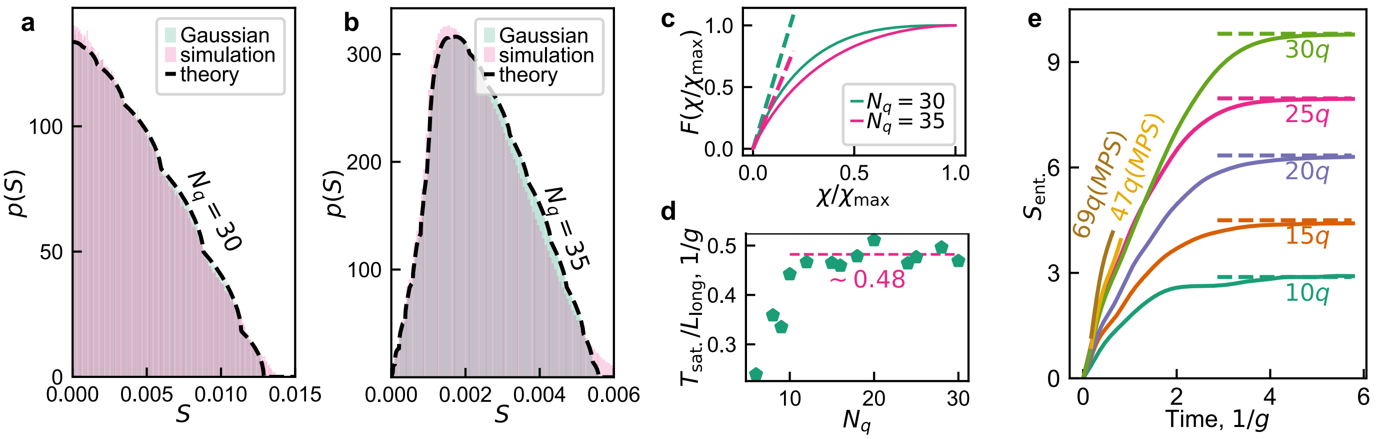

Fig. S11. **Entanglement properties of the simulated wave functions. a,b,** Distribution of Schmidt values in a 30- and 35-qubit systems, where we compare the quarter-circle theory, random Gaussian ensemble, and wave functions obtained in the simulation of the effective spin Hamiltonian. **c,** State fidelity as a function of the bond dimension cutoff. The dashed lines show linear slope $F \leqslant \zeta(N_{\rm q})(\chi/\chi_{\max}) \approx 4\sqrt{2}(\chi/\chi_{\max})$. **d,** The time it takes for the system to build 90 % of the maximum entanglement at given system size, divided by the longest dimension of the rectangle $L_{\rm long}$. The linear fit at $\sim 0.48 \times (1/g)$ indicates convergence $t_{\rm sat.} \sim (1/2g) \times L_{\rm long}$. **e,** Entanglement entropy as a function of time in classical ideal simulations for various system sizes using state-vector and MPS approaches. The MPS simulations are stopped upon saturation of the bond dimension. The dashed lines show entanglement entropy of typical half-filled states estimated using Eq. (23).

$0 \leqslant x < L_x$, $0 \leqslant y < L_y$, and $L_x \leqslant L_y$, i. e., the system is cut into two halves along the shortest direction.

Consider a system with M particles and $N = L + R$ sites, with a wave function $|\psi\rangle$ written in the full 2^N-element basis. A matrix element of the reduced density matrix (RDM) of the left-hand side reads

$$(\hat{\rho}_L)_{ij} = \left(\mathrm{Tr}_R\, [|\psi\rangle\langle\psi|]\right)_{ij} = \sum_{r=0}^{2^R-1} \psi^*_{i,r}\psi_{j,r}, \tag{16}$$

where $i(j)$ and r enumerate basis states in the L or R subsystem, respectively. Since the total number of excitations M is fixed, the matrix elements are non-zero only for (i, j) satisfying

$$\mathrm{Ham}[i] + \mathrm{Ham}[r] = \mathrm{Ham}[j] + \mathrm{Ham}[r] = M, \tag{17}$$

where $\mathrm{Ham}[k]$ counts the number of excitations in a bitstring k (Hamming weight). Therefore, $\mathrm{Ham}[i] = \mathrm{Ham}[j] = M - \mathrm{Ham}[r]$, and the reduced total density matrix has a block-diagonal form

$$\hat{\rho}_L = \bigoplus_{M_L=0}^{\min(L,M)} \hat{\rho}_{L,M_L}, \tag{18}$$

where $0 \leqslant M_L \leqslant \min(L, M)$ is the number of excitations in the left-hand side of the system. At half-filling, the total number of Schmidt values is 2^L. To obtain the Schmidt spectrum, we perform eigendecomposition of all partial density matrices $\hat{\rho}_{L,M_L}$.

We now consider typical distributions of the Schmidt values in the $U(1)$–conserving case at infinite temperature. In a generic case (without particle number conservation), the RDM is irreducible. If the system is divided into the left and right parts with L and R sites and the effective Hilbert space sizes D_L, D_R ($D_L D_R = 2^N$), the Schmidt values follow the generalized quarter-circle distribution[63]

$$p(S) = \frac{D}{\pi}\frac{\sqrt{(\lambda_+^2/D_L - S^2)(S^2 - \lambda_-^2/D_L)}}{\lambda S}, \tag{19}$$

where $\lambda = D_L/D_R$, $\lambda_\pm = 1 \pm \sqrt{\lambda}$, and $\lambda_-/\sqrt{D_L} \leqslant S \leqslant \lambda_+/\sqrt{D_L}$.

In contrast, in the $U(1)$–symmetric case, the Schmidt values follow the quarter-circle distribution independently in each M_L–block of the RDM[64]. The $U(1)$–constrained quarter-circle distribution of the Schmidt values S_i reads

$$p(S) = \sum_{M_L=0}^{L} \begin{cases} 0, & S \notin [\lambda_-^{M_L}, \lambda_+^{M_L}], \\ p_{M_L}(S), & S \in [\lambda_-^{M_L}, \lambda_+^{M_L}], \end{cases} \text{, with} \tag{20}$$

$$p_{M_L(S)} = \frac{D}{S\pi} \times \sqrt{\left[\left(\lambda_+^{M_L}\right)^2 - S^2\right]\left[S^2 - \left(\lambda_-^{M_L}\right)^2\right]},$$

where $D = \binom{N}{M}$ is the full Hilbert space dimension, $D_L^{M_L} = \binom{L}{M_L}$ is the number of ways to put M_L particles in the left part, $D_R^{M_L} = \binom{R}{M-M_L}$ is the number of ways to put $(M - M_L)$ particles in the right part, and $\lambda_\pm^{M_L} = (1 \pm \sqrt{\lambda^{M_L}})\sqrt{D_R^{M_L}/D}$ with $\lambda^{M_L} = D_L^{M_L}/D_R^{M_L}$.

In Fig. S11a,b, we show the Schmidt values distribution of the 30 and 35-qubit systems with 15 and 18 photons, respectively. The distributions are obtained (i) using the exact simulation of the system Hamiltonian, (ii) from Schmidt-decomposing a wave function with random complex Gaussian entries $\psi_i \sim \mathcal{N}(0,1) + i\mathcal{N}(0,1)$, and (iii) applying the equation Eq. (20), which accurately describes the Schmidt value distributions. We conclude that the time evolution with the Hamiltonian $H_{\rm s}$ generates typical states, in the sense of $U(1)$–constrained quarter-circle Schmidt value distribution.

The complexity of a MPS encoding of time-evolved states is determined by the fraction $\chi/\chi_{\max}$, where $\chi_{\max}$ is the maximum possible number of Schmidt values given Hilbert space sizes, required to represent a wave function with a given fidelity $\mathcal{F}$. In Fig. S11c, integrating the distribution Eq. (20), we plot $\mathcal{F}(\chi/\chi_{\max})$. In the half-filled case, the derivative at zero is given by

$$\mathcal{F} \leqslant 4\frac{\chi}{\chi_{\max}} \frac{2^L \sqrt{\binom{L}{L/2}\binom{R}{R/2}}}{\binom{N}{N/2}} = 4\frac{\chi}{\chi_{\max}}\left(\sqrt{2} + \mathcal{O}(1/N)\right), \tag{21}$$

which differs by a factor $\sqrt{2}$ from the generic case with no particle conservation[38]. This formula will be used below to derive bounds on complexity of the experimentally realized states.

2. Entanglement entropy

In Fig. S11d,e we consider the entanglement entropy growth with time at various system sizes. The dashed lines indicate the maximum entanglement entropy at a given system size at half-filling with $U(1)$ conservation. Using Eq. (20), these bounds can be computed as[65]:

$$S_{\text{ent.}} = \sum_{M_L=0}^{L} \frac{D_R^{M_L} D_L^{M_L}}{D}\left[\log\left(\frac{D}{D_R^{M_L}}\right) - \frac{\lambda^{M_L}}{2}\right]. \tag{22}$$

Assuming $L = R$ and the half-filled case with even $M = (L+R)/2$, it can be further simplified (replacing summation over M_L with a Gaussian integral):

$$S_{\text{ent.}}^{U(1)} = \frac{N}{2}\log 2 - \frac{1}{2}\log 2 - \frac{1}{4} + \mathcal{O}(1/N). \tag{23}$$

This expression is different from $S_{\text{ent.}}^{\text{generic}} = \log\sqrt{D} - 1/2 = (N/2)\log 2 - 1/2 = S_{\text{ent.}}^{U(1)} + 0.097$, obtained for an irreducible RDM. Fig. S11e shows that the entanglement entropy of the simulated wave functions approaches $S_{\text{ent.}}^{U(1)}$ at long times.

Fig. S11d shows the ratio of the time it takes the system to build 90 % of the maximum entanglement entropy, $t_{\text{sat.}}$, to the longest direction of the rectangle L_{long}. Since the maximum entropy is proportional to the total system volume, $\max S_{\text{ent.}} \propto L_{\text{long}}L_{\text{short}}$, and the rate of entanglement generation at early times is proportional to the length of the shortest cut, $dS_{\text{ent.}}/dt \propto L_{\text{short}}$, we expect $t_{\text{sat.}} = \alpha L_{\text{long}}$. Indeed, Fig. S11d shows that the ratio $t_{\text{sat.}}/L_{\text{long}}$ saturates at around $\alpha \approx (1/2g)$ in the simulation with $g/(2\pi) \approx 10\,\text{MHz}$.

3. Log-negativity

For noisy dynamics on a quantum device, it is customary to compute the log-negativity

$$\mathcal{E}_{\text{N}}(\psi) = \log_2 \| |\psi\rangle\langle\psi|^{T_A} \|_1, \tag{24}$$

where $|\psi\rangle\langle\psi|^{T_A}$ is a partially-transposed (in a subsystem A) density matrix of a pure system with the wave function $|\psi\rangle$. Here, we focus on a bipartition of a system into equal-sized regions L and R. Given log-negativity of a pure state, it is possible to bound the mixed-state entanglement of a mixed state $\hat{\rho}$ (quantified by log-negativity of a mixed state $\mathcal{E}_{\text{N}}(\hat{\rho})$) using the pure-state log-negativity and the notion of fidelity. In Ref. [41], it was shown that, if the desired pure state $|\psi\rangle$ is an eigenstate of the mixed-state density matrix $\hat{\rho}$ with an eigenvalue F, the following bound holds

$$\mathcal{E}_{\text{N}}(\hat{\rho}) \geqslant \mathcal{E}_{\text{N}}(|\psi\rangle) + \log_2 F, \tag{25}$$

where $F = \langle\psi|\hat{\rho}|\psi\rangle$ is the state fidelity[41]. Notably, in an actual experiment, the pure state will not be an exact eigenstate of the density matrix. Nevertheless, Ref. [41] has shown that the bound Eq. (25) holds for generic time evolution and generic noise sources. Log-negativity of a pure state $|\psi\rangle$ is equivalent to the Rényi–1/2 entropy and could be computed as

$$\mathcal{E}_{\text{N}}(\psi) = 2\log_2 \sum_{i=0}^{2^L-1} S_i, \tag{26}$$

where S_i are the Schmidt values.

In the $U(1)$–symmetric case, we obtain

$$\sum_{i=0}^{2^L-1} S_i \to \int_0^{+\infty} \mathrm{d}S\, S\, p(S) = \frac{D}{3\pi}\sum_{M_L=0}^{L} \lambda_+^{M_L} \times \tag{27}$$
$$\times\left[\left[\left(\lambda_-^{M_L}\right)^2 + \left(\lambda_+^{M_L}\right)^2\right]\tilde{E}(q) - 2\left(\lambda_-^{M_L}\right)^2 \tilde{F}(q)\right],$$

where $q = \sqrt{1 - \left(\lambda_-^{M_L}/\lambda_+^{M_L}\right)^2}$, and $\tilde{F}, \tilde{E}$ are complete elliptic integrals of the first and the second kind, respectively. In a system with $L = R = N/2$ and even $M = (L+R)/2$, the expressions simplify to $\lambda_-^{M_L} = 0$, $\lambda_+^{M_L} = 2\sqrt{D_R^{M_L}/D}$ and $q = 1$ and the sum reads:

$$\sum_{i=0}^{2^L-1} S_i \to \frac{8}{3\pi}\sum_{M_L=0}^{L} \frac{\left(D_R^{M_L}\right)^{3/2}}{D^{1/2}} = \tag{28}$$
$$= \frac{2^{3/4}\times 8}{3\sqrt{3\pi}} 2^{N/4}\left(1 + \mathcal{O}(1/N)\right),$$

and we obtain

$$\mathcal{E}_N^{U(1)} = N/2 + \log_2 \frac{2^{3/2}}{3}\frac{64}{9\pi^2} + \mathcal{O}(1/N) = \tag{29}$$
$$= \frac{S_{\text{ent.}}^{U(1)}}{\log 2} + \frac{1}{2} + \frac{1}{4\log 2} + \log_2\frac{2^{3/2}}{3}\frac{64}{9\pi^2} + \mathcal{O}(1/N) \approx$$
$$\approx \frac{S_{\text{ent.}}^{U(1)}}{\log 2} + 0.303.$$

In contrast, a system with the Hilbert space D where the

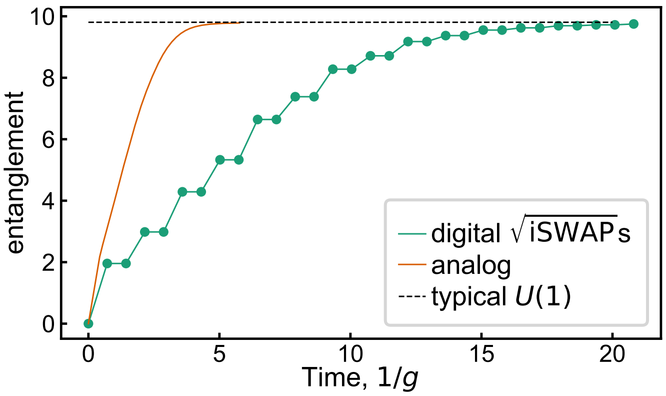

Fig. S12. **Comparison of entanglement growth in the digital and analog cases.** The points show entanglement entropy in the digital case after application of each layer in the ABCD gate pattern. The black dashed line shows the typical entanglement in the $U(1)$ case of a 30-qubit system obtained using Eq. (23).

RDM does not form blocks, the log-negativity reads

$$\mathcal{E}_{\rm N}^{\rm generic} = \log_2 \sqrt{D} + \log_2 \frac{64}{9\pi^2} = \quad (30)$$
$$= \frac{S_{\rm ent.}^{\rm generic}}{\log 2} + \frac{1}{2\log 2} + \log_2 \frac{64}{9\pi^2} \approx \frac{S_{\rm ent.}^{\rm generic}}{\log 2} + 0.248.$$

We use the expression for log-negativity in its $U(1)$–conserving (Eq. (29)) and generic (Eq. (30)) forms in the main text to approximate the maximum mixed-state entanglement entropies obtained in different experiments.

In the main text, Figure 2d, on the x-axis we plot the effective system size defined as

$$N_{\rm q}^{\rm eff} = 2S_{\rm ent}/\log 2 + 1/\log 2, \quad (31)$$

such that $N_{\rm q}^{\rm eff} = N_{\rm q}$ for a generic system. In turn, in the y-axis, we plot $\mathcal{E}_{\rm N}$ against $N_{\rm q}^{\rm eff} = 2S_{\rm ent}/\log 2 + 1/\log 2$ and draw an ideal line. However, for the $U(1)$–conserving and generic cases, the correction to $\mathcal{E}_{\rm N} = S_{\rm ent.}/\log 2 + C$ is slightly different ($C_{U(1)} = 0.303$ against $C_{\rm generic} = 0.248$, respectively). For simplicity, to plot the ideal line, we use the mean value $\bar{C}$ between these two corrections and plot $\mathcal{E}_{\rm N} = (1/2)N_{\rm q}^{\rm eff} - 1/(2\log 2) + \bar{C} \approx (1/2)N_{\rm q}^{\rm eff} - 0.449$.

4. Rényi-2 entropy

The Rényi-2 entropy could be experimentally obtained via randomized purity measurements[66]. Similarly to the Rényi-1/2 entropy, in the $U(1)$–symmetric case, entanglement of a typical quarter-circle state reads

$$\sum_{i=0}^{2^L-1} S_i^4 \to \int_0^{+\infty} {\rm d}S\, S^4\, p(S) = \quad (32)$$
$$\frac{D}{32} \sum_{M_L=0}^{L} \left[\left(\lambda_+^{M_L}\right)^2 - \left(\lambda_-^{M_L}\right)^2\right] \times \left[\left(\lambda_+^{M_L}\right)^2 + \left(\lambda_-^{M_L}\right)^2\right].$$

Considering a system with $L = R$ and even $M = (L + R)/2$, we obtain the $\log_2$–based expression

$$S_{\text{Rényi-2 ent.}}^{U(1)} = (N/2) + \log_2 \frac{\sqrt{3}}{4} + \mathcal{O}(1/N). \quad (33)$$

5. Entanglement growth in digital and analog cases

In this subsection, we compare the rates of the bipartite von Neumann entropy growth in the digital and analog settings. We consider a two-dimensional 5×6 lattice with open boundary conditions and cut the lattice into two 15-qubit parts along the shorter direction. In the analog case, we simulate the pure XY-model with the coupling constant g. In the digital case, we consider a period-4 ABCD pattern (left, up, right, down) of $\sqrt{\text{iSWAP}}$ gates. Applying a layer of such gates requires time $t = (\pi/4)(1/g)$. We can therefore put analog and digital simulations on the same time-axis. The result is shown in Fig. S12. We observe that both entropies reach the bound set by the quarter-circle theory, with the analog version being nearly 4 times as fast.

S11. Rényi-2 entanglement entropy

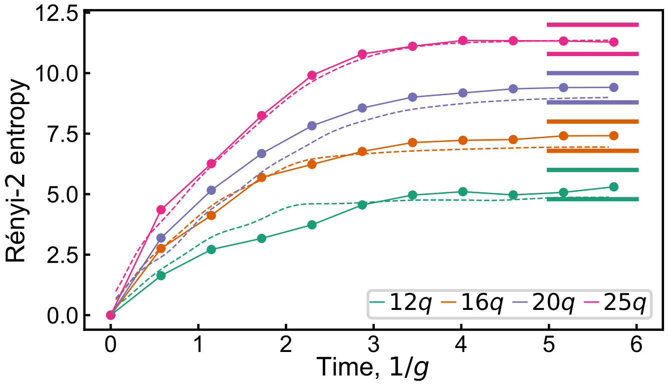

Fig. S13. **Measurement of the bipartite Rényi-2 entanglement entropy.** Solid lines are the experimental results, and the dashed lines are fidelity-adjusted simulation results. For each system size, the lower bold line on the right denotes entropy of a typical state with $U(1)$ conservation given by Eq. (33), and the upper bold line corresponds to the maximally-depolarized state at this system size.

1. Measurement

In order to measure the entanglement entropy without fully reconstructing the density matrices, we utilize randomized measurement techniques[66], and the results are shown in Fig. S13. To probe various properties of the prepared quantum states, we apply M sets of single-qubit Clifford gates prior to measurement to all qubits in our system, and repeat the measurement to obtain K shots.

Using this protocol we obtain $M \times K$ bitstrings, which we use to compute entanglement entropy. The state purity $P = \mathrm{tr}(\hat{\rho}^2)$ is estimated using

$$P = \frac{2^N}{M} \sum_{m=1}^{M} \sum_{s,s'}^{2^N} P(s)P(s')(-2)^{-D[s,s']}\,, \tag{34}$$

where N corresponds to the number of qubits in the (sub)system, and $D[s, s']$ is the Hamming distance between the two bitstrings s and s'. In order to mitigate the bias in the estimation of purity arising from finite samples, we utilize jackknife resampling to obtain the unbiased purity estimate $\hat{P}_{\mathrm{unbiased}}$ from the measured value P using the following formula

$$P_{\mathrm{unbiased}} = \frac{K}{K-1}\hat{P} - \frac{2^N}{K-1}\,. \tag{35}$$

Finally, we calculate the second Rényi entropy

$$S_2(\hat{\rho}) = -\log_2(P_{\mathrm{unbiased}}) \tag{36}$$

to characterize the entanglement entropy of the (sub)system.

In order to probe the entanglement entropy of volume-law (typical) subsystems of up to size 12 in our system, we use $M = 50$ different pre-measurement unitaries, with $K = 10^6$ shots each. For a typical state, the probability of measuring a given bitstring scales as 2^{-N}. Therefore, the accurate reconstruction of the probability distribution requires many shots. The bitstring probability distribution does not vary by much when measured in different bases, hence, we only need to use a small M. In contrast, when probing area-law states we need larger M whilst requiring fewer shots. In our experiments, we use $M = 1000$ pre-measurement unitaries with $K = 5 \times 10^4$ shots for such states. In order to accurately probe the entanglement scaling crossover from area- to volume-law as a function of the number of excitations in the initial state n_0, we use $M = 1000$ and $K = 5 \times 10^4$ for states with $n_0 < 8$, and $M = 50$ and $K = 10^6$ for states with $n \geqslant 8$.

2. Numerics

To match the experimental data, we consider finite fidelity-corrections to the simulated Schmidt spectrum. To this end, we write the full system density matrix as

$$\hat{\rho} = F|\psi\rangle\langle\psi| + (1-F)\frac{\mathrm{Id}}{D}, \tag{37}$$

assuming the global depolarizing channel, where Id is the identity matrix. If the system is split into the left and right parts with sizes L and R, the RDM of the left-hand side reads

$$\hat{\rho}_L = \bigotimes_{M_L=0}^{\min(L,M)} \left(F\hat{\rho}_{L,M} + \frac{1-F}{D} D_R^{M_L}\mathrm{Id} \right), \tag{38}$$

where the direct product runs over the number of excitations in the left part, similarly to Eq. (18). Randomized purity measurements compute

$$\mathrm{Tr}\,\hat{\rho}_L^2 = \sum_{M_L=0}^{\min(L,M)} \mathrm{Tr}\left[F\hat{\rho}_{L,M} + \frac{1-F}{D} D_R^{M_L}\mathrm{Id} \right]^2, \tag{39}$$

where $\hat{\rho}_{L,M}$ is the perfect system density matrix. This expression allows us to estimate the effect of finite fidelity on the Rényi entropy estimations, $-\log_2 \mathrm{Tr}\,\hat{\rho}_L^2$. In Fig. S13, we show the corrected result, using the $F(N_{\mathrm{q}}, tg)$ dependence obtained in Section S9.

S12. Classical computational complexity

In this section, we address the classical simulation complexity of the full 69-qubit chip using tensor-network contractions and MPS simulations.

1. Tensor network contraction

In a tensor network contraction approach, a circuit is represented in the form of elementary tensors (gates) with legs that need to be contracted[67–69]. In case of XEB benchmarking, we are interested in a particular amplitude $\langle s|\psi\rangle$, where $|\psi\rangle$ is the simulated wave function and s is a bitstring sampled on a quantum device. If $\hat{U}(t)$ represents a unitary evolution performed on a quantum device starting from an initial state $|s_0\rangle$, we need to compute scalars of the form $\langle s|\hat{U}(t)|s_0\rangle$. While tensor contraction methods are directly applicable to the problems of random circuit sampling[70–76], to study analog evolution with tensor network contraction methods, we first find an efficient digital circuit that represents the time evolution. For a fair comparison, the circuit should be chosen to minimize its contraction cost.

We assume that the gates in a digital circuit representing the time evolution can be collected, layer-by-layer, into projected entangled-pair operators (PEPOs). The resulting tensor network is shown in Fig. S14a. For concreteness, we specifically assume that the time evolution can be written in terms of PEPOs with virtual bond dimension $\chi = 2$, and that these PEPOs are maximally efficient in terms of generating entanglement. Given the virtual bond dimension, a single PEPO application generates $\Delta S_{\mathrm{ent.}} = L_{\mathrm{short}} \log 2$ entanglement entropy across the minimal cut that divides the system in half. Since the maximum entanglement entropy is $S_{\mathrm{ent.}}^{\max} = (1/2)L_{\mathrm{short}}L_{\mathrm{long}} \log 2$, it takes at least $N_{\mathrm{PEPOs}} = (1/2)L_{\mathrm{long}}$ layers of PEPO application to saturate the entanglement entropy. Note that considering larger bond dimension of the form $\chi = 2^k$ does not change this consideration. Indeed, it would generate the same amount of entanglement as k PEPOs with $\chi = 2$, and also could be written as consecutive application of k such PEPOs.

As shown in Sec. S10, the entanglement entropy saturates in $(1/2g) \times L_{\mathrm{long}}$. Therefore, a single PEPO application generates the amount of entropy which corresponds

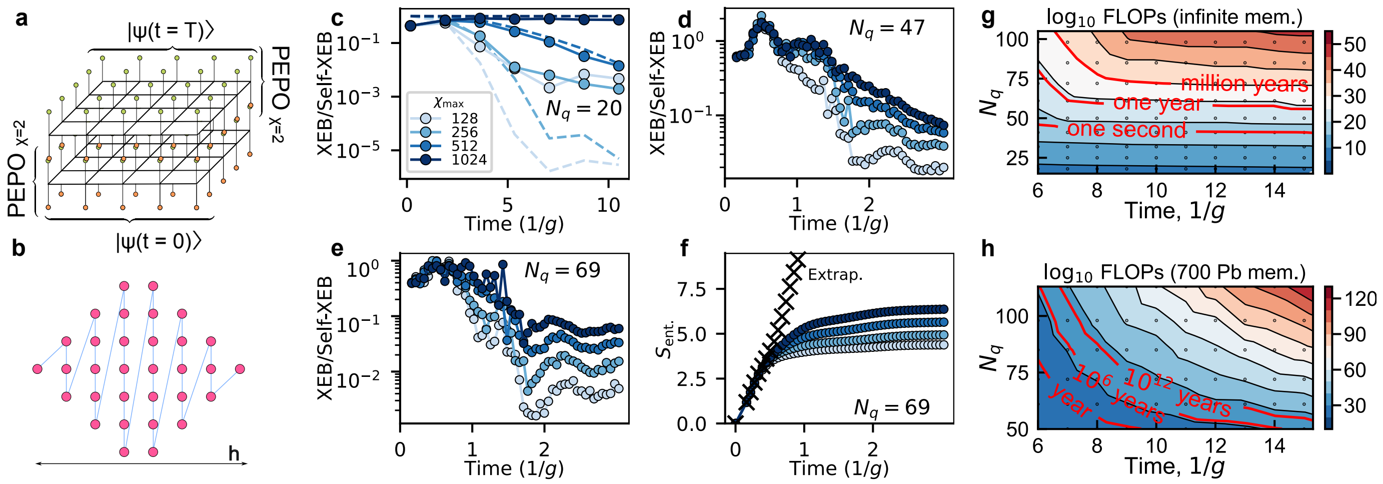

Fig. S14. **Classical computation complexity.** **a,** Representation of the hypothetical most efficient tensor network representation of the time evolution. Here, we show application of two PEPO layers to a wave function $|\psi(0)\rangle$ to obtain a wave function at time t, $|\psi(t)\rangle$, **b,** A family of Sycamore-like geometries with varied height h considered in this panel for complexity analysis. The blue line shows a typical MPS "snake" used to build an MPS wave function. **c,** The ratio of XEB for $N_\mathrm{q} = 20$, computed from experimental data and MPS simulations, to self-XEB, computed by sampling bitstrings from the MPS. Dashed lines show the fidelity of MPS wave functions relative to the exact time evolution. When the classical results are exact ($\chi = 1024$), XEB/Self-XEB is a measure of experimental fidelity; at smaller χ, we find a lower ratio. The x-axis gives the time in inverse coupling strength, taking into account weaker coupling during ramps. **d,** XEB/Self-XEB for $N_\mathrm{q} = 47$. MPS fidelity at $\chi = 1024$ is high until time approximately $1/g$, then decays rapidly. Self-XEB at time $3/g$ extrapolates in $1/\chi$ to around 2, so that the PTD would not yet be achieved. **e,** The $N_\mathrm{q} = 69$ system. **f,** Growth of entanglement in the MPS simulation of the 69-qubit system. At short times, an extrapolation in $1/\log(\chi)$ gives the expected linear growth of entanglement. **g,** Computational complexity of the TN-contraction algorithm, assuming no memory constraints, and **h,** assuming using the full Frontier hard drive of 700 PB[77]. The red lines convert the FLOPs count in Frontier time assuming its peak 2 exaFLOPs performance[77].

to the evolution time of a single cycle $(1/g)$ defined in the main text. This conversion allows us to estimate the contraction costs, considering a set of Sycamore-like tilted square geometries shown in Fig. S14b.

In Fig. S14g, we show the FLOPs count required to contract a tensor network with N_q qubits and a given number of analog cycles, assuming infinite memory. Here, we do not account for finite fidelity of the device. The best contraction is found using a simulated annealing algorithm repeated with numerous attempts.

Finally, in Fig. S14h, we show complexity assuming usage of the whole Frontier hard drive (700 PB)[77] and ignoring communication costs. Finite memory limits the size of a maximum tensor that could be stored in memory during the computation. To avoid having a larger tensor, some edges in the tensor network are projected and then the final result is summed over all 2^{N_sliced} possible choices of N_sliced sliced variables[68,71,78]. To account for finite fidelity of the simulation, we only consider the respective fraction of the sliced variables' combinations[68]. We observe that contraction of a circuit representing evolution on a 69-qubit chip until the maximum-entanglement time would require $\mathcal{O}(10^6)$ years on the Frontier supercomputer assuming its peak 2 exaFLOPs performance[77].

2. *Matrix product states—complexity bounds*

In this subsection, we estimate the complexity of sampling bitstring form the distribution $|\langle s|\hat{U}(t)|s_0\rangle|^2$, using matrix product states (MPS). We consider the "MPS snake" site encoding, as shown in Fig. S14b. In this section, the entanglement and corresponding MPS bond dimension, χ, are considered for a half-system cut. In addition to the experimental fidelity $\mathcal{F}_\mathrm{exp}$, we consider fidelity $\mathcal{F}_{\chi_\mathrm{max}}$ of the time-evolved MPS with the peak bond dimension at most χ_max. Given the total time-evolution length t, we need to estimate the minimum peak bond dimension χ_max such that (i): $\mathcal{F}_{\chi_\mathrm{max}}(t) \geqslant \mathcal{F}_\mathrm{exp}(t)$. This requirement only at time t is weaker than demanding (ii): $\mathcal{F}_{\chi_\mathrm{max}}(\tau) \geqslant \mathcal{F}_\mathrm{exp}(\tau)$ for all $0 \leqslant \tau \leqslant t$, and therefore (i) gives a lower bound on (ii).

We choose the total evolution time t such that satisfying criterion (i) with MPS is as hard as possible. In the experiment, entanglement entropy builds up linearly and saturates at $t_\mathrm{sat.} \sim L_\mathrm{long}/(2g)$, as shown in Fig. S11. At $t > t_\mathrm{sat.}$, $F_\mathrm{exp.}(t)$ decays exponentially, which makes $t = t_\mathrm{sat.}$ a natural choice. However, a single bitstring amplitude $\langle s|\hat{U}(t)|s_0\rangle$ can be obtained by first obtaining $\hat{U}(t/2)|s_0\rangle$ and $\hat{U}(-t/2)|s\rangle$, and then finding their overlap, so we may only time-evolve MPS to half-time. Therefore, we choose $t = 2t_\mathrm{sat.}$. On a 69-qubit system with $L_\mathrm{long} = 8$, $2t_\mathrm{sat.} \sim 8/g$. As shown in Section. S9, the extrapolated experimental fidelity at this time is $\mathcal{F}_\mathrm{exp}(2t_\mathrm{sat.}) \approx 0.4$. To achieve such fidelity at $t/2 = t_\mathrm{sat.}$, an MPS would employ the bond dimension of $\chi_\mathrm{max} \approx 1.7 \times 10^9$. This would require around $50\times$ the total hard drive of Frontier for the largest individual tensors. This estimate did not account for the $U(1)$ conservation.

With the $U(1)$ symmetry, the MPS tensors have a block

structure[79]. We find numerically for the 69 qubit system that at bond dimensions of 2^7, 2^8, 2^9, and 2^{10}, at $t = 3/g$ the required memory is multiplied by ≈ 0.20, 0.19, 0.18, and 0.17, respectively. A linear extrapolation in $1/\log(\chi)$ shows that the memory requirement for large bond dimensions is reduced by $m_{U(1)} \sim 10$, therefore the largest MPS tensors would still exceed the Frontier hard drive.

Finally, let us assume no memory constraint and estimate the pure FLOPs requirement to perform such MPS simulation, which is dominated by the singular value decomposition (SVD). For a real $n \times n$ matrix, a SVD takes $\mathcal{O}(n^3)$ operations, and the MPS compressions involve truncation of $2\chi \times 2\chi$ complex matrices due to inclusion of the physical dimension of 2 on each site, leading to the $64\chi^3$ FLOPs requirement per SVD. The $U(1)$ symmetry reduces the cost by a factor $m^2_{U(1)}$. We will conservatively only include the cost of SVD at the central cut with largest bond dimension, ignoring decompositions at other cuts.

Assuming a single cycle requires N_{Trotter} steps, we get the total number of SVD decompositions $N_{\text{SVD}} = 4N_{\text{Trotter}}$. We assume that the entanglement increases linearly during the simulation up to time $t_{\text{sat.}}$, and therefore the required bond dimension grows at step k as $\chi(k) \sim (\chi_{\max})^{k/N_{\text{SVD}}}$. The required FLOPs estimate reads

$$\text{FLOPs} = \frac{64}{m^2_{U(1)}} \sum_{k=0}^{N_{\text{SVD}}} \left((\chi_{\max})^{k/N_{\text{SVD}}}\right)^3 \sim \sim \frac{64}{m^2_{U(1)}} \frac{N_{\text{SVD}}}{3\log\chi_{\max}} \chi^3_{\max}. \quad (40)$$

Maintaining low Trotter error requires at least 5 sweeps per cycle, which gives FLOPs $\sim 10^{27}$ (with $\chi_{\max} \approx 1.7 \times 10^9$), which would take $0.5 \times 10^9\,\text{s} = 16\,\text{years}$ on Frontier assuming its peak performance of 2 exaFLOPs and ignoring any communication costs.

Importantly, in this procedure with forward and backward evolution, final bitstrings s will be chosen from i.i.d. and will not follow the PTD. Rejection sampling [80] corrects for this at the cost of an extra $\mathcal{O}(10)$ sampling overhead. Thus the effective time per bitstring for MPS is lower-bounded by 160 years.

3. *Matrix product states — practical demonstration*

We run MPS time evolution simulations for the system sizes $N_\text{q} = 20$, 47, and 69; we show the results in Fig. S14c-f[1]. The MPS sites are ordered as shown in Fig. S14b. We account for the $U(1)$ conservation and construct a near-optimal matrix product operator (MPO) using deparallelization and delinearization[81]. We time-evolve using the two-site time-dependent variational principle (TDVP)[82,83], using time steps $\delta t \leqslant 0.05/g$ so that time discretization is not a significant source of error. We simulate the full time-dependent experimental procedure, including ramps and plateau time. For each system size, we consider bond dimensions $\chi \in \{128, 256, 512, 1024\}$, with truncation after each local TDVP step. To track the MPS fidelity, we either (i) record truncation error at each step, or (ii) compute the fidelities between the same-time states with the various bond dimensions.

For $N_\text{q} = 20$, where $\chi = 1024$ yields no truncation, and (ii) is exact, the results are shown as dashed lines in Fig. S14c. Similarly, for all considered system sizes, MPS simulations at our largest bond dimensions maintain high fidelity until times $\sim 1/g$, followed by an exponential decay with the rate higher than the experimental (see Sec. S9).

We consider the ratio of XEB to self-XEB, gives an estimate of fidelity (see Sec. S13 2). To obtain linear XEB, we perform MPS evolution until time t with bond dimension χ, and compute

$$\text{XEB} = \frac{D}{M} \sum_{x \sim p_{\text{meas}}(x,t)} p_{\text{MPS}}(x,t) - 1 \quad (41)$$

with x being the experimental bitstrings, and

$$\text{self-XEB} = \frac{D}{M} \sum_{x \sim p_{\text{MPS}}} p_{\text{MPS}}(x,t) - 1, \quad (42)$$

where x are sampled from an MPS[84,85], and M is the total number of samples.

At $N_\text{q} = 20$, experimental fidelity is above 0.5 for times up to $10/g$, and the MPS simulations at $\chi = 1024$ are exact. Indeed, in Fig. S14c, we observe XEB/self-XEB being close to 1 for $\chi = 1024$. Furthermore, for $\chi = 512$, XEB/self-XEB is close to the MPS fidelity; since the experimental fidelity is high, this is consistent with XEB/self-XEB giving an approximate relative fidelity between the experiment and the MPS. When the MPS fidelity is lower, the XEB/self-XEB ratio provides no clear information, as plots with $\chi = 128$ and 256 show.

With these insights, we can use MPS to examine XEB/self-XEB for systems of 47 and 69 qubits. Generally, rapidly-growing entropy does not allow for reliable fidelity decays extrapolations. However, for $N_\text{q} = 47, 69$, shown in Figs. S14d-e, the MPS fidelity for the largest bond dimension remains close to 1 until plateau time $\approx 1/g$. In this regime the MPS is not significantly truncated (there is a clear convergence towards larger bond dimensions), and thus the ratio XEB/self-XEB gives an approximate handle on the experimental fidelity (at these times, the self-XEB is still of order 100, so the distribution of bitstrings is far from PTD, making the connection only approximate). Therefore, XEB/self-XEB ~ 1 in Fig. S14d-e verifies that the quantum device is behaving similarly to the simulation, and that the experimental fidelity remains high at least within the first cycle. At longer times, although the MPS fidelity decays rapidly, the XEB/self-XEB is converging with increasing bond dimension. In principle, an extrapolation in bond dimension could allow a quantitative estimate of fidelity decay rate at longer times.

---

[1] Similar results were found for $N_\text{q} = 30$ and 60.

In addition to fidelity estimates, at short times the entanglement entropy between two halves of the system can be computed. In Fig. S14f we show the entanglement vs time at different bond dimensions for the full 69-qubit system. We find the expected near-linear growth in entanglement at short times before reaching the plateau. We linearly extrapolate in $1/\log(\chi)$ to infinite bond dimension, showing that linear growth of entanglement would continue as expected if we used larger χ. The short-time entanglement entropies for $\chi = 1024$ with $N_\mathrm{q} = 47$ and 69 are also shown in Fig. S11e.

S13. Bitstring distributions and XEB

In this section, we provide evidence that the simulated wave functions to a large degree follow the Porter-Thomas distribution (PTD) $\Pr(p) = De^{-pD}$, where D is the system Hilbert space dimension. The PTD is a key element in benchmarking the state fidelity using XEB[38]. We will also study the applicability of the recently suggested protocols to remove the non-PTD corrections from the distribution of bitstrings in a wave function obtained in analog evolution.

1. Distributions and self-XEB

We give the summary of results in Fig. S15. In Fig. S15a, we plot the bitstring probability distributions of the wave functions obtained in half-filled simulation of the $g/(2\pi) \approx 10$ MHz and $g/(2\pi) \approx 20$ MHz cases for system sizes varying between 16 and 35 qubits. The wave functions are taken, respectively, after 300 and 150 ns of time evolution, corresponding to 18 cycles. The Hilbert space dimension $D = \binom{N_\mathrm{q}}{N_\mathrm{q}/2}$ accounts for the particle number conservation. As the system size increases, we observe slight deviation of the bitstring distributions from the PTD, which becomes worse in the $g/(2\pi) \approx 20$ MHz case, where non-XY terms have greater magnitude. The degree of agreement with the PTD could be quantified by self-XEB $D\sum_s p^2(s) - 1$, where $p^2(s)$ is the simulated probability of a bitstring s, and the sum runs over the whole Hilbert space. In case of a PTD, self-XEB is unity. Generally, self-XEB could vary from 0 for a fully-depolarized state, to $D-1$ for a fully-localized state. In Fig. S15b, we show self-XEB as a function of time for different system sizes and $g/(2\pi) \approx 10, 20$ MHz. It reaches $\mathcal{O}(1)$ after a short time of $t \leqslant 4/g$. In Fig. S15c, we show the absolute deviation of self-XEB from 1, δ as a function of time. We observe that, as seen in Fig. S15a, the $g/(2\pi) \approx 20$ MHz simulation has a larger deviation (up to $\delta \sim 0.03$) from the PTD, as compared to the $g/(2\pi) \approx 10$ MHz case, where PTD is nearly-reached.

2. XEB benchmarking of fidelity

In this section, we will discuss benchmarking of the state fidelity using XEB. To this end, we use a simple model for the system density matrix

$$\hat{\rho} = \Phi|\psi_{\mathrm{exp.}}\rangle\langle\psi_{\mathrm{exp.}}| + (1-\Phi)\frac{\mathrm{Id}}{D}, \tag{43}$$

where Id is the identity matrix representing the global depolarizing noise channel, $|\psi_{\mathrm{exp.}}\rangle$ is the true wave function that describes the device evolution, and Φ represents fidelity of the time evolution with respect to non-coherent errors. Additionally, due to the calibration imperfections, the simulated wave function $|\psi_{\mathrm{sim.}}\rangle$ is different from $|\psi_{\mathrm{exp.}}\rangle$, which is realized on the chip. We could express the simulated wave function as

$$|\psi_{\mathrm{sim.}}\rangle = \sqrt{f}|\psi_{\mathrm{exp.}}\rangle + \sqrt{1-f}|\psi_\perp\rangle, \tag{44}$$

where $\langle\psi_{\mathrm{exp.}}|\psi_\perp\rangle = 0$ and $f = |\langle\psi_{\mathrm{exp.}}|\psi_{\mathrm{sim.}}\rangle|^2$ represents fidelity with respect to coherent errors. With these definitions, the experimental probabilities read

$$p_{\mathrm{exp.}}(s) = \langle s|\hat{\rho}|s\rangle = p_s\Phi + (1-\Phi)/D \tag{45}$$

with $p_s = |\psi_{\mathrm{exp.}}(s)|^2$, and the simulated probabilities read

$$p_{\mathrm{sim.}}(s) = |\psi_{\mathrm{sim.}}(s)|^2 = p_s f + \\ +2\mathrm{Re}\left[\sqrt{f(1-f)}\psi_{\mathrm{exp.}}(s)\psi_\perp^*(s)\right] + (1-f)p_{\perp,s} \tag{46}$$

with $p_{\perp,s} = |\psi_\perp(s)|^2$. The XEB then reads

$$\mathrm{XEB} = D\sum_s p_{\mathrm{exp.}}(s)p_{\mathrm{sim.}}(s) - 1 = D\sum_s \left[f\Phi p_s^2 + \Phi p_s\left(2\mathrm{Re}\left[\sqrt{f(1-f)}\psi_{\mathrm{exp.}}(s)\psi_\perp^*(s)\right] + (1-f)p_{\perp,s}\right) + \\ + \left[\frac{1-\Phi}{D}\left(p_s f + 2\mathrm{Re}\left[\sqrt{f(1-f)}\psi_{\mathrm{exp.}}(s)\psi_\perp^*(s)\right] + (1-f)p_{\perp,s}\right)\right]. \tag{47}$$

We work out these terms and then combine them into the final expression. First, we note the simple relations such as

$$D\sum_s p_s^2 = \text{self-XEB} + 1, \quad \sum_s p_{\perp,s} = \sum_s p_s = 1, \tag{48}$$

$$D\sum_s p_s p_{\perp,s} = D^2\,\mathbb{E}\,p_s \times \mathbb{E}\,p_{\perp,s} = 1, \tag{49}$$

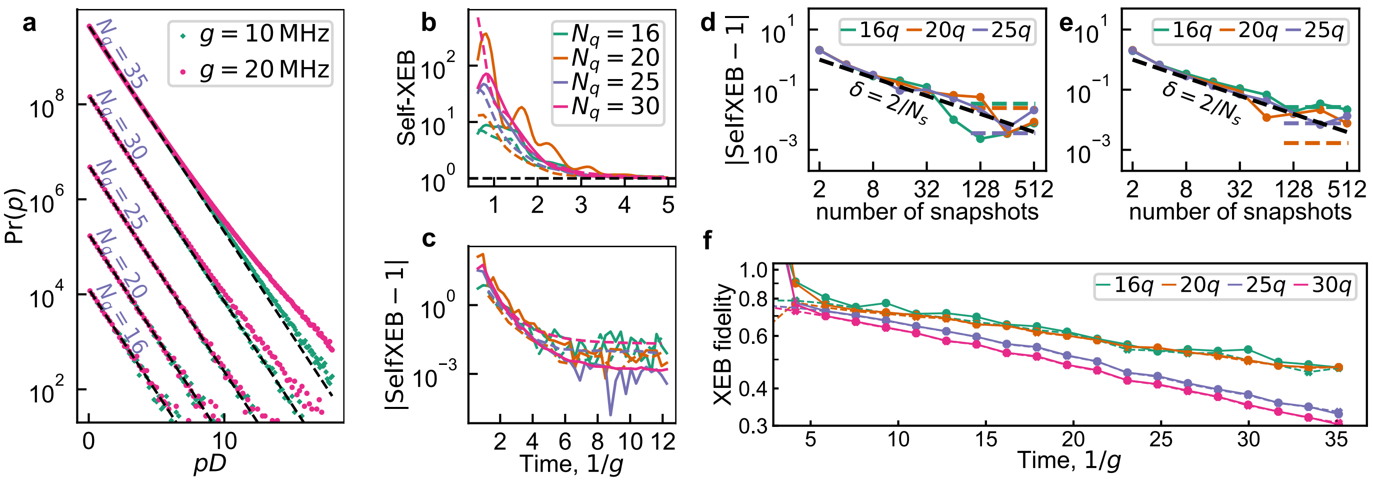

Fig. S15. **Bitstring distributions of simulated wave functions.** **a,** The probabilities distribution of the simulated wave functions for $g/(2\pi) \approx 10\,\text{MHz}$ and $g/(2\pi) \approx 20\,\text{MHz}$. The black dashed lines correspond to the ideal Porter-Thomas distribution. **b,** Convergence of self-XEB in analog evolution to 1 for $g/(2\pi) \approx 10\,\text{MHz}$ (bold) and $g/(2\pi) \approx 20\,\text{MHz}$ (dashed). **c,** Time-dependence of the imperfection $\delta = |\text{self-XEB} - 1|$. **d, e,** The imperfection after $p_{\text{avg.}}(s_i)$-renormalization as a function of the number of snapshots N_s for $g/(2\pi) \approx 10\,\text{MHz}$ and $g \approx 20\,\text{MHz}$, respectively. The black dashed line shows the theoretical prediction $\delta = 2/N_s + \mathcal{O}(1/N_s^2)$, while the colored dashed line show the imperfections for different system sizes without the renormalization. **f,** Linear XEB fidelity as a function of time measured in $1/g$ for various system sizes for $g/(2\pi) \approx 10\,\text{MHz}$, the dataset used in the main text. The solid lines show XEB estimator F before renormalization, while dashed lines show the XEB estimator $\tilde{F}$ after renormalization.

$$\mathbb{E}\sqrt{p_s p_{\perp,s}}\cos\theta_s = 0,\ \mathbb{E}\left(\sqrt{p_s p_{\perp,s}}\cos\theta_s\right)^2 = 1/(2D^2), \tag{50}$$

similarly

$$\mathbb{E}\sqrt{p_s^3 p_{\perp,s}}\cos\theta_s = 0,\ \mathbb{E}\left(\sqrt{p_s^3 p_{\perp,s}}\cos\theta_s\right)^2 = 3/D^4. \tag{51}$$

Under these assumptions, the full XEB expression simplifies to

$$\begin{aligned} \text{XEB} = f\Phi(\text{self-XEB}+1) + \Phi(1-f) + (1-\Phi)f + \\ +(1-f)(1-\Phi) = f\Phi\text{self-XEB} = F \times \text{self-XEB}, \end{aligned} \tag{52}$$

where we defined $F = f\Phi$, fidelity reflecting coherent and non-coherent errors. Therefore, we obtain

$$\text{XEB}/\text{self-XEB} = F, \tag{53}$$

which coincides with the estimator F_e from Ref. [48].

3. Correlated probabilities in analog dynamics

Above, we treated p_s and $p_{\perp,s}$ as uncorrelated variables. However, the probability distributions p_s and $p_{\perp,s}$ obtained from analog dynamics with conservation laws may retain a large degree of correlation, which would introduce corrections to the relation Eq. (53), since $\mathbb{E}\,p_s p_{\perp,s} \neq \mathbb{E}\,p_s\,\mathbb{E}\,p_{\perp,s}$. A symptom of these residual correlations is that the p_s distribution has self-XEB $\neq 1$, i.e., imperfect agreement with the PTD.

The information about the underlying analog dynamics and conservation laws is contained in the time-averaged probability distribution

$$p_{\text{avg.}}(s) = \lim_{T\to\infty}\frac{1}{T}\int_0^T dt\,|\psi_{\text{sim.}}(t,s)|^2, \tag{54}$$

where $|\psi_{\text{sim.}}(t,s)|^2$ is the s-bitstring probability for the simulated wave function at time t. Having obtained $p_{\text{avg.}}(s)$ by averaging over N_s wave function snapshots, we could renormalize any probability distribution as $p_s \to p_s/(p_{\text{avg.}}(s)D)$, and re-weight the expectation values as $\mathbb{E}\,X_s = \sum_s p_{\text{avg.}}(s)X_s$, thus removing the prevalence of certain bitstrings due to the details of the particular Hamiltonian dynamics implemented by the analog device[48].

In the limit of large sample N_s, the renormalized self-XEB $= D\sum_s p_{\text{avg.}}(s)(p_s/p_{\text{avg.}}(s))^2 - 1$ converges to 1, and the non-universal deviations from the PTD are removed. However, estimation of $p_{\text{avg.}}$ with finite N_s leads to sampling noise, which for insufficient N_s could make the imperfection $\delta = |\text{self-XEB} - 1|$ actually larger than if we did not perform the re-weighting at all. To estimate the required sample size in order to achieve a given error level δ, we consider a model case where the renormalization is applied to the PTD. We assume that $p_{\text{avg.}}(s)$ is estimated over N_s samples sufficiently separated in time, such that all $p_{t,s} = |\psi_{\text{sim.}}(t,s)|^2$ could be seen as uncorrelated random variables drawn from PTD: $p_{t,s} \sim De^{-p_{t,s}D}$. The inverse mean $1/p_{\text{avg.}}$ of these N_s samples is distributed as

$$1/p_{\text{avg.}} \sim \frac{N_s D p_{\text{avg.}}^2 (N_s D p_{\text{avg.}})^{N_s-1}}{(N_s-1)!}e^{-N_s D p_{\text{avg.}}}, \tag{55}$$

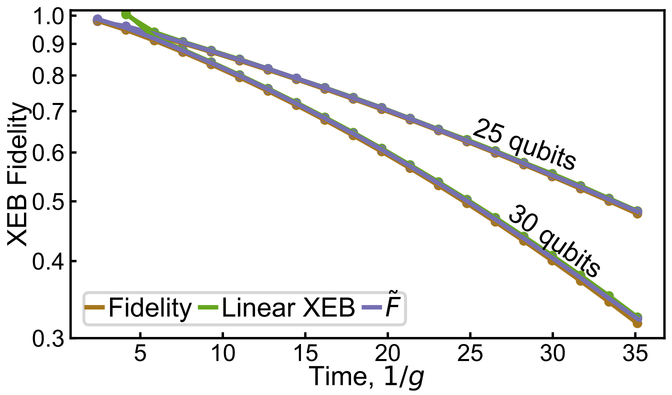

Fig. S16. **Representation of fidelity by XEB fidelity estimators** The curves represent fidelity, linear XEB and the $\tilde{F}$ estimator between the "experimental" and "simulated" states, which differ by coherent errors in the Hamiltonian. As a reference state, we pick 25- and 30-qubit wavefunctions considered in the main text. To compute $p_{\rm avg.}$, we average over 501 wave function snapshots from 100 to 600 ns with a step of 1 ns.

giving $\langle 1/[Dp_{\rm avg.}(s)]\rangle = 1 + 1/N_s + \mathcal{O}(1/N_s^2)$. Therefore, self-XEB $= 1 + 2/N_s + \mathcal{O}(1/N_s^2)$ for the case of the PTD renormalized with a finite sample N_s. As a result, for a non-PT distribution with $|\text{self-XEB} - 1| = \delta$ before re-weighting, $|\text{self-XEB} - 1|$ will be reduced by the re-weighting procedure if $N_s \leqslant 2/\delta$. We verify this scaling in Fig. S15d,e, considering renormalization of the analog distributions using a range of sample sizes N_s. Since the distributions produced in our analog dynamics are already close to the PTD, δ follows a $2/N_s$ trend robustly for both $g/(2\pi) \approx 10, 20\,\text{MHz}$. The crossing with the non-renormalized value of δ happens at $N_s \sim 2/\delta$, and the self-XEB improves afterwards.

Having considered improvement of the analog distributions in terms of self-XEB, we now consider application of this technique to the fidelity estimator F = XEB/self-XEB. If both experimental and theoretical distributions follow PTD, the renormalized version reads

$$\tilde{F} = \frac{\sum_s p_{\rm exp}(s) p_{\rm sim}(s)/p_{\rm avg}(s) - 1}{\text{self-XEB}} = \tag{56}$$
$$= F\left(1 - \frac{1}{N_s}\right) + \frac{1}{N_s} + \mathcal{O}(N_s^{-2}),$$

which means that the finite sample size N_s would lead to overestimation of fidelity. Therefore, considering simulated $|\text{self-XEB} - 1| \sim 0.01$, we apply this renormalization technique to the main dataset of the main text, averaging over 500 wave function instances to obtain $p_{\rm avg.}(s)$. After renormalization, we compute the renormalized fidelity $\tilde{F}$, and the results are shown in Fig. S15f. We observe that the naive estimator F (solid) agrees with the renormalized estimator $\tilde{F}$ (dashed). Therefore, we conclude that removing Hamiltonian-specific bias by means of $p_{\rm avg.}(s)$ renormalization leaves the XEB decay rate unchanged, which demonstrates that our XEB proxy for fidelity is not affected by significant correlations between the probabilities p_s and $p_{\perp,s}$.

4. Agreement between fidelity and estimators

In this section, we show that the fidelity estimators used in this work faithfully represent the state fidelity. Here, we only consider the fidelity loss due to coherent errors, i. e., caused by calibration or modelling imperfections, which are shown to dominate the error in Sec. S6 . To this end, we consider (i) the wave functions $\psi_{\rm opt.}$ at the optimal global shifts $\mathrm{d}g^{nXX} = \mathrm{d}g^{XnX} = -20\,\text{kHz}$ used in the main text at $g/(2\pi) \approx 10\,\text{MHz}$ to benchmark experimental fidelity, and (ii) the wave functions $\psi_{\rm bare.}$ without these shifts applied. We treat the $\psi_{\rm opt.}$ wave functions as the ground truth $\psi_{\rm exp.}$, and $\psi_{\rm bare.}$ as our simulation $\psi_{\rm sim.}$.

This allows us to evaluate how well linear XEB and the renormalized estimator, $\tilde{F}$ (Eq. (56)) represent fidelity $|\langle\psi_{\rm exp.}|\psi_{\rm sim.}\rangle|^2$. The results are shown in Fig. S16. In agreement with our previous findings of the near-PTD shape of our probability distributions, we obtain good representation of fidelity by both naive linear XEB and its renormalized version Eq. (56).

[59] Hernández, M. Chebyshev's approximation algorithms and applications. Comput. Math. Appl. **41**, 433–445 (2001).
[60] Yuan, S., De Raedt, H. & Katsnelson, M. I. Modeling electronic structure and transport properties of graphene with resonant scattering centers. Phys. Rev. B **82** (2010).
[61] Westerhout, T. lattice-symmetries: A package for working with quantum many-body bases. J. Open Source Softw. **6**, 3537 (2021).
[62] Cai, Y., Tong, Y. & Preskill, J. Stochastic error cancellation in analog quantum simulation (2023). 2311.14818.
[63] Marčenko, V. A. & Pastur, L. A. Distribution of eigenvalues for some sets of random matrices. Mat. Sb. **1**, 457 (1967).
[64] Cheng, Y., Patil, R., Zhang, Y., Rigol, M. & Hackl, L. Typical entanglement entropy in systems with particle-number conservation (2023). URL https://arxiv.org/abs/2310.19862.
[65] Lin, R. Exact analytical relation between the entropies and the dominant eigenvalue of random reduced density matrices (2022). URL https://arxiv.org/abs/2204.01754.
[66] Elben, A. et al. The randomized measurement toolbox. Nat. Rev. Phys. **5**, 9–24 (2023).
[67] Markov, I. L. & Shi, Y. Simulating quantum computation by contracting tensor networks. SIAM Journal on Computing **38**, 963–981 (2008). https://doi.org/10.1137/050644756.
[68] Villalonga, B. et al. A flexible high-performance simulator for verifying and benchmarking quantum circuits implemented on real hardware. npj Quantum Information **5**, 86 (2019).
[69] Gray, J. & Kourtis, S. Hyper-optimized tensor network contraction. Quantum **5**, 410 (2021).

[70] Boixo, S., Isakov, S. V., Smelyanskiy, V. N. & Neven, H. Simulation of low-depth quantum circuits as complex undirected graphical models (2018). 1712.05384.

[71] Chen, J., Zhang, F., Huang, C., Newman, M. & Shi, Y. Classical simulation of intermediate-size quantum circuits (2018). 1805.01450.

[72] Pednault, E. et al. Pareto-efficient quantum circuit simulation using tensor contraction deferral (2020). 1710.05867.

[73] Huang, C. et al. Classical simulation of quantum supremacy circuits (2020). 2005.06787.

[74] Kalachev, G., Panteleev, P. & Yung, M.-H. Multi-tensor contraction for xeb verification of quantum circuits (2022). 2108.05665.

[75] Pan, F. & Zhang, P. Simulation of quantum circuits using the big-batch tensor network method. Phys. Rev. Lett. **128**, 030501 (2022).

[76] Pan, F., Chen, K. & Zhang, P. Solving the sampling problem of the sycamore quantum circuits. Phys. Rev. Lett. **129**, 090502 (2022).

[77] Atchley, S. et al. Frontier: Exploring exascale. In Proceedings of the Conference for HPC, SC '23 (Association for Computing Machinery, New York, NY, USA, 2023). URL https://doi.org/10.1145/3581784.3607089.

[78] Huang, C. et al. Efficient parallelization of tensor network contraction for simulating quantum computation. Nature Computational Science **1**, 578–587 (2021).

[79] Singh, S., Pfeifer, R. N. C. & Vidal, G. Tensor network states and algorithms in the presence of a global u(1) symmetry. Phys. Rev. B **83**, 115125 (2011).

[80] Markov, I. L., Fatima, A., Isakov, S. V. & Boixo, S. Quantum supremacy is both closer and farther than it appears (2018). 1807.10749.

[81] Hubig, C., McCulloch, I. P. & Schollwöck, U. Generic construction of efficient matrix product operators. Phys. Rev. B **95**, 035129 (2017).

[82] Haegeman, J., Lubich, C., Oseledets, I., Vandereycken, B. & Verstraete, F. Unifying time evolution and optimization with matrix product states. Phys. Rev. B **94**, 165116 (2016).

[83] Paeckel, S. et al. Time-evolution methods for matrix-product states. Annals of Physics **411**, 167998 (2019).

[84] Stoudenmire, E. M. & White, S. R. Minimally entangled typical thermal state algorithms. New Journal of Physics **12**, 055026 (2010).

[85] Ferris, A. J. & Vidal, G. Perfect sampling with unitary tensor networks. Phys. Rev. B **85**, 165146 (2012).
